# Circulating miR-206 and miR-1246 as Markers in the Early Diagnosis of Lung Cancer in Patients with Chronic Obstructive Pulmonary Disease

**DOI:** 10.3390/ijms241512437

**Published:** 2023-08-04

**Authors:** Elizabeth Córdoba-Lanús, Angélica Domínguez de-Barros, Alexis Oliva, Delia Mayato, Francisca Gonzalvo, Ana Remírez-Sanz, Javier J. Zulueta, Bartolomé Celli, Ciro Casanova

**Affiliations:** 1 Department of Internal Medicine, Dermatology and Psychiatry, Universidad de La Laguna, 38071 San Cristóbal de La Laguna, Spain; casanovaciro@gmail.com; 2 Instituto Universitario de Enfermedades Tropicales y Salud Pública de Canarias, Universidad de La Laguna, 38296 San Cristóbal de La Laguna, Spain; angelica4arealejos@gmail.com (A.D.d.-B.); amoliva@ull.edu.es (A.O.); 3 Centro de Investigación Biomédica en Red de Enfermedades Infecciosas (CIBERINFEC), Instituto de Salud Carlos III, 28029 Madrid, Spain; 4 Department of Pharmaceutical Technology, Universidad de La Laguna, 38206 Santa Cruz de Tenerife, Spain; 5 Pulmonary Department-Research Unit, Hospital Universitario Nuestra Señora de Candelaria, 38010 Santa Cruz de Tenerife, Spain; deliamayato@gmail.com (D.M.); franciscagonzalvo@hotmail.com (F.G.); 6 CIMA, Centro de Investigación Médica Aplicada, Universidad de Navarra, 31008 Pamplona, Spain; aremirez@unav.es; 7 Navarra Institute for Health Research (IdISNA), 31008 Pamplona, Spain; jzulueta@unav.es; 8 Pulmonary, Critical Care and Sleep Medicine Division, Mount Sinai Morningside Hospital, New York, NY 10029, USA; 9 Centro de Investigación Biomédica en Red de Cáncer (CIBERONC), 28029 Madrid, Spain; 10 Pulmonary Critical Care Medicine Division, Brigham and Women’s Hospital, Harvard Medical School, Boston, MA 02115, USA; bcelli@copdnet.org; 11 Centro de Investigación Biomédica en Red de Enfermedades Respiratorias (CIBERES), Instituto de Salud Carlos III, 28029 Madrid, Spain

**Keywords:** lung cancer, COPD, circulating miRNAs, biomarkers

## Abstract

Lung cancer (LC) is the most common cause of cancer death, with 75% of cases being diagnosed in late stages. This study aimed to determine potential miRNAs as biomarkers for the early detection of LC in chronic obstructive pulmonary disease (COPD) cases. Ninety-nine patients were included, with registered clinical and lung function parameters followed for 6 years. miRNAs were determined in 16 serum samples from COPD patients (four with LC and four controls) by next generation sequencing (NGS) at LC diagnosis and 3 years before. The validation by qPCR was performed in 33 COPD-LC patients and 66 controls at the two time points. Over 170 miRNAs (≥10 TPM) were identified; among these, miR-224-5p, miR-206, miR-194-5p, and miR-1246 were significantly dysregulated (*p* < 0.001) in COPD-LC 3 years before LC diagnosis when compared to the controls. The validation showed that miR-1246 and miR-206 were differentially expressed in COPD patients who developed LC three years before (*p* = 0.035 and *p* = 0.028, respectively). The in silico enrichment analysis showed miR-1246 and miR-206 to be linked to gene mediators in various signaling pathways related to cancer. Our study demonstrated that miR-1246 and miR-206 have potential value as non-invasive biomarkers of early LC detection in COPD patients who could benefit from screening programs.

## 1. Introduction

Lung cancer is the second most prevalent cancer and the most common cause of cancer death [1], with more than 2.2 million new cases of lung cancer in 2020 and 1.80 million deaths globally.

Non-small-cell lung cancer (NSCLC), which accounts for 85% of lung cancers [2], has a poor prognosis (5-year overall survival rate < 15%), and the main contributing factor to this is that more than 75% of lung cancer cases are diagnosed in late stages because of the lack of screening programs for persons at risk [2]. Low-dose computed tomography (LDCT) is the lung cancer screening (LCS) tool most useful in detecting LC at its early stages [3]. Although it is effective in reducing mortality, it has some drawbacks such as the high rate of false positive results that drives the performance of invasive procedures and the overall high cost for the health system.

Early detection offers clear advantages in treating and monitoring the disease by improving cure rates, so alternative approaches to improve the identification of those cases are needed. Patients who suffer from chronic obstructive pulmonary disease (COPD), secondary to tobacco use, are at increased risk of developing LC, with an incidence that is 2–5 times that of smokers without COPD [4,5]. Genetics and epigenetics seem to play important roles as contributors to LC development [5,6,7]. Therefore, the finding of non-invasive, reliable biomarkers that can help increase the probability of earlier LC detection in individuals at increased risk, as COPD patients are, seems like a fertile area to explore.

At present, cancer-related biomarkers are not specific to tissues and have poor early diagnosis and prognostic value; therefore, they cannot be used as targeted therapy for cancers. MicroRNAs (miRNAs) have emerged as good potential biomarkers of early diagnosis, the monitoring of progression, and the treatment of many cancers [8]. miRNAs are short non-coding RNAs, 19 to 25 nucleotides in length, involved in virtually all cellular processes, including development, apoptosis, and cell cycle regulation [9,10]. Moreover, miRNAs have shown great stability in different specimens and tissues [11]. After their biogenesis, mature miRNAs interact with certain messenger RNAs (mRNAs) in the cytoplasm during post-transcriptional regulation in an induced RNA-silencing multiprotein complex (RISC) [11]. The dysregulation of miRNAs caused by either genetic or epigenetic mechanisms has been described in various diseases ranging from cancer [12] to autoimmune diseases [13], lung diseases [14], and viral infections [15]. In addition, it has been proposed that gene therapy using miRNAs might be used to block the progression of cancers [16] by inhibiting the overexpression of oncogenic miRNAs or replacing those that are effective on tumor-suppressive genes. Thus, miRNAs can serve as either biomarkers or therapeutic targets in cancer, such as NSCLC.

Several studies have shown that a miRNA profile can be used to discriminate subjects with NSCLC from controls [17,18,19]. However, there is scarce information about miRNAs’ usefulness as early markers of LC in patients at risk, and, as far as we know, not one report in COPD patients exists. This study was performed in a well-phenotyped COPD cohort with a follow-up of more than 10 years, with the main objective of characterizing a miRNA profile capable of early detection of those patients developing LC during the years of observation.

## 2. Results

### 2.1. miRNA Screening Study

A total of eight individuals (COPD patients with LC, and COPD patients who did not develop LC during follow-up as controls) were included in the screening study with biological samples in two timelines of analysis (at the time of LC diagnosis and 3 years before). The cases and controls were male, age-matched (mean age of 64 years) and presented similar smoking habits. The main clinical characteristics of these individuals are shown in Tables S1 and S2. Most of the COPD patients (90%) had moderate airway obstruction. Smokers with COPD and LC had higher FEV_1_ and lower K_CO_ values than COPD controls, but a similar proportion of emphysema was visualized by CT scan. LC patients’ histological subtypes were non-small-cell lung cancer (NSCLC) (75% adenocarcinoma, 25% squamous carcinoma).

**Figure 1:**
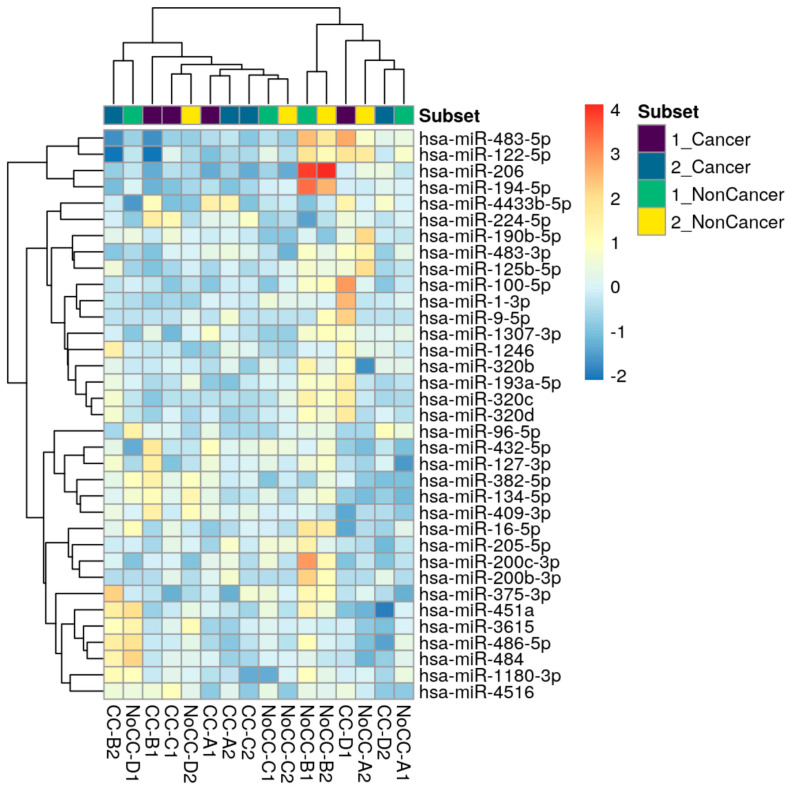
Top genes with the highest variance across samples were selected for hierarchical clustering. Each row represents one gene, and each column represents one sample. The color represents the difference of the count value to the row mean (−2 to 4).

**Figure 2:**
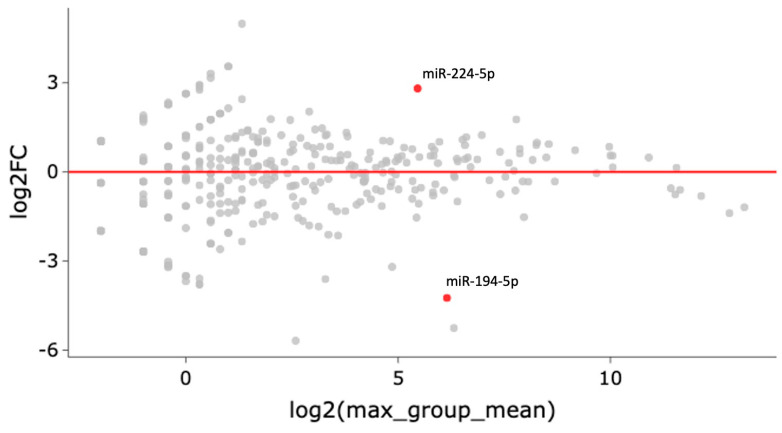
Each gene’s fold change (FC) is plotted against its mean expression among all samples three years before LC diagnosis. All significantly differentially expressed genes are marked in red. Significant changes are defined as *p*-value < 0.001, FDR < 0.01, and Log2FC > 2.

Over one hundred and seventy miRNAs (≥10 TPM) were identified by next-generation sequencing (NGS) in the 16 samples analyzed (Figure 1). Between these, hsa-miR-224-5p was found significantly upregulated and hsa-miR-194-5p was found significantly downregulated in COPD patients with LC three years before LC diagnosis when compared to COPD cases that did not develop LC during follow-up (log2FC = 2.81 and log2FC = −4.24, respectively; *p* < 0.0001, FDR < 0.001) (Figure 2). hsa-miR-206 was found significantly dysregulated in COPD individuals 3 years before these patients had a LC diagnosis (log2FC = −5.25, *p* < 0.001), although the correction for multiple testing was FDR > 0.01 (Table 1).

At the time of LC diagnosis, patients with COPD who developed LC presented dysregulated hsa-miR-194-5p and hsa-miR-206 expression levels when compared to controls (*p* = 0.0036 and *p* < 0.0001, respectively), while hsa-miR-1246 maintained increased levels of expression (*p* = 0.0032, but FDR > 0.05) (Table 1).

### 2.2. Validation Study

The validation of the miRNA expression analysis was performed on all 99 participants included in the study for the two timeline points: at the time of LC diagnosis (33 LC cases, 66 controls), and three years before (21 LC cases and 30 controls). The main clinical characteristics of cases and controls are shown in Table 2 and Table 3. The patients were primarily men (85%), with a mean age of 63 years, and heavy smokers. The patients with COPD and LC had higher FEV_1_ and lower K_CO_ values than the COPD controls, but a similar proportion of emphysema. The tumor histological subtypes present in the LC patients were mostly non-small-cell lung cancer (NSCLC) (61% adenocarcinoma, 27% squamous carcinoma, 3% microcytic carcinoma, and two cases of undifferentiated carcinoma).

**Figure 3:**
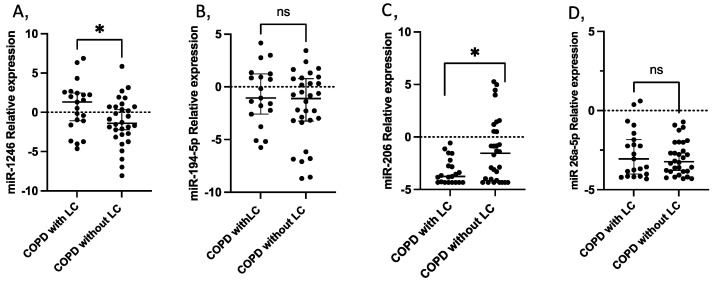
Circulating miRNA expression levels in serum samples from 21 COPD patients with LC three years before diagnosis compared with 30 COPD without LC as controls. (**A**) miR-1246 (*p* = 0.035); (**B**) miR-194-5p; (**C**) miR-206 (*p* = 0.028); (**D**) miR-26-5p. Lines represent the median with an interquartile range. * *p* < 0.05; ns: non-significant.

In the validation experiments, miR-224 was expressed in less than 85% of the studied population, so it was excluded for further analysis. miR-1246 was overexpressed in patients with COPD who developed LC 3 years before LC diagnosis when compared to COPD controls who did not develop LC (log2FC = 2.63, *p* = 0.032) (Figure 3). A significant difference in miR-1246 expression was also found at the time of LC diagnosis (log2FC = −4.79, *p* = 0.022) (Figure S1). miR-206 was also found dysregulated between cases and controls 3 years before (log2FC = −2.205, *p* = 0.027) (Figure 3), and maintained at the time of LC diagnosis (log2FC = −1.624, *p* = 0.023) (Figure S1).

The expression of miR-194-5p did not differ significantly between the study groups in either of the two timelines.

**Figure 4:**
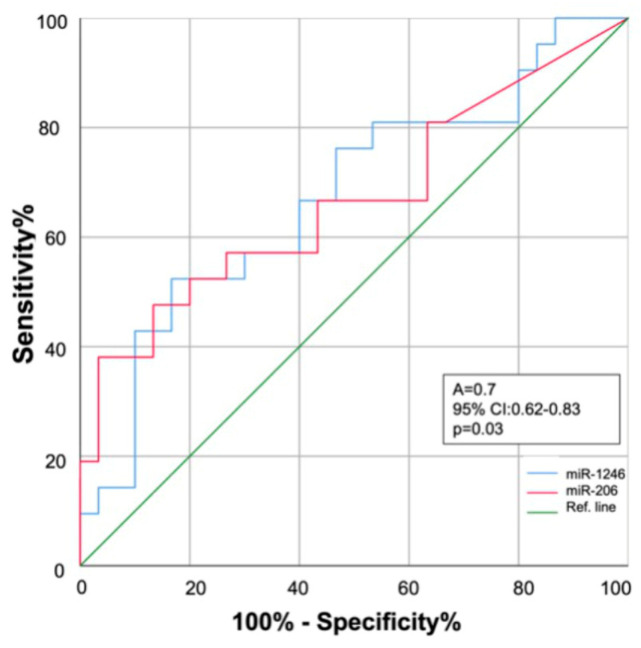
Receiver operating characteristics (ROC) curve analysis. Diagnostic performance of miR-1246 (blue line) and miR-206 (red line) for early discrimination of COPD patients who will develop lung cancer within the next three years. AUC: area under the curve; 95% CI: 95% confidence interval.

ROC curve analysis showed that miR-1246 and miR-206 expression in serum could discriminate between COPD controls and patients with COPD who will develop LC within the next three years (AUC: 0.68 (95% confidence interval (CI) = 0.62~0.85), *p* = 0.035; and AUC: 0.68 (95% CI = 0.63~0.83), *p* = 0.030, respectively) (Figure 4).

### 2.3. Clinical Relations

The levels of expression of miR-26a-5p and miR-194-5p in COPD patients who would go on to develop LC within the next 3 years correlated significantly with the levels of arterial oxygen PaO_2_ (r = 0.74, *p* = 0.035, and r = 0.97, *p* < 0.001, respectively), although this measure was only available in a small sample of individuals (Figure S2). No relationship was found between any other miRNA expression in COPD individuals with LC and other clinical variables.

### 2.4. Functional Annotation Analysis and Gene Target Prediction

Our functional analysis, through the KEGG pathways, revealed miR-1246 to be significantly associated with target genes enriched in several functions and signaling pathways. The most significant ones are reported in Table 4A: the viral carcinogenesis, the apoptosis pathway, the TP53 signaling pathway, and the central carbon metabolism in cancer. On the other hand, miR-206 was shown to be significantly associated with the enriched target genes detailed in Table 4B: glycosphingolipid biosynthesis, miRNAs in cancer, proteoglycans in cancer, and transcriptional misregulation in cancer.

The GO analysis of the collection of target genes of miR-1246 together with miR-206 was enriched in several functions, including the cellular nitrogen metabolic process, the biosynthetic process, the protein modification process, gene expression, the cellular component or protein assembly, membrane organization, and the mitotic cell cycle (Table S3).

## 3. Discussion

The presence of chronic obstructive pulmonary disease (COPD) is an important risk factor for developing lung cancer. Considerable effort is being made to identify biomarkers that, in combination with conventional tests, can help identify individuals more likely to develop LC and candidates for close monitoring and early diagnosis of LC. This study is the first to describe the altered expression of two circulating miRNAs, hsa-miR1246 and hsa-miRNA-206, that can be detected in the serum samples of patients with COPD up to three years before lung cancer diagnosis and could help identify subjects at high risk for LC development who are likely to benefit from screening programs.

### 3.1. miR-1246 and Predictive Risk of Lung Cancer

In our study, miR-1246 was found overexpressed in individuals with COPD and LC three years before diagnosis, and this differential expression was maintained at the time of diagnosis when compared to COPD controls who did not develop LC. Moreover, we could detect increased levels of this miRNA in patients who developed LC in relation to the presence of emphysema, a well-known risk factor of LC [6]. Our findings are in accordance with Yang et al., who, in a small group of patients, identified miR-1246 to be the most upregulated miRNA in the serum of patients with LC when compared to healthy controls [20]. However, this study was only conducted at the time of diagnosis, thus not informing its value as a toll for early screening for LC. Interestingly, these authors also reported that this miRNA increased the migration and invasiveness of A549 lung cancer cells. Specifically, they observed that E-cadherin expression decreased and vimentin and transforming growth factor-β expression increased, indicating the possible role of miR-1246 in the Wnt/-βcatenin pathway and its relation to cancer progression and metastasis. Similarly, in the study by Kim et al., miR-1246 was found to be overexpressed in sphere-forming cells, and an anti-miR-1246 strategy was effectively shown to suppress the proliferation, sphere-formation, colony-forming ability, and invasiveness of cancerous cells [21]. In addition, other authors have documented a role for miR-1246 in the progression of non-small-cell lung cancer [22].

Our study’s functional in silico analysis suggests that miR-1246 may be linked to gene mediators in various signaling pathways related to cancer. When exploring the KEGG pathway analysis in relation to the miR-1246 target genes, it was enriched, for example, in the P53 signaling pathway. Also, miR-1246 has been identified as a novel p53 target miRNA, as shown by the study of Liao et al., who reported p53 to inhibit DYRK1A expression through the induction of miR-1246 in non-small-cell lung cancer cells [23]. Several studies indicate that P53, a typical tumor suppressor gene, is involved in NSCLC development and progression [24]. BCL2L2 is a predicted target of miR-1246. It this context, increases in Bcl-2 protein expression have been shown to contribute to the development of a wide variety of human cancers, including lung cancer, and may act as a resistance factor against several anticancer agents [25]. Another predicted target of miR-1246 is HER4 (ERBB4,) a tyrosine kinase receptor. EGFR is a plasma membrane glycoprotein that belongs to this family of receptors. EGFR dimerization may result in cancer cell proliferation, the inhibition of apoptosis, invasion, metastasis, and tumor-induced neovascularization [26].

### 3.2. miRNA-206 and Predictive Risk of Lung Cancer

Another important finding was that miR-206 dysregulation can be detected early in patients with COPD who will develop lung cancer three years before diagnosis. The low expression of miR-206 is related to lung cancer invasion and metastasis [27,28]. miR-1 expression, which is related to miR-206, was found to be decreased in lung cancers [29]. It was reported that the treatment with HDAC as an inhibitor of lung cancer cells could induce the expression of repressed miR-1 by the downregulation of oncogenes such as *MET*, *PIM1*, *FOXP1*, and *HDAC4* [29]. *MET* is a common target in three of the pathways detected for miR-206 in the in silico analysis of the present study: miRNAs in cancer, proteoglycans in cancer, and transcriptional misregulation in cancer.

Interestingly, Yang et al. found that forced miR-206 expression restored gefitinib sensitivity in IL6-induced gefitinib-resistant EGFR-mutant lung cancer cells by inhibiting the IL6/JAK1/STAT3 pathway [30]. Furthermore, studies performed on NSCLC in A549 cells showed that the miR-206 antagomir therapy decreased tumor volume and the formation of intra-tumoral capillary tubes, and increased apoptosis by blocking the 14-3-3zeta/STAT3/HIF-1alpha/VEGF signaling pathway [31]. miR-206 has been reported to function as a tumor metastasis suppressor in different type of cancers, including colon and gastric cancers [32,33].

### 3.3. miRNAs and Their Relation to Pulmonary Function

We found a strong correlation between miR-26a-5p and miR-194-5p expression and partial oxygen arterial tension (PaO_2_) in COPD patients who went on to develop LC within the next 3 years. Other studies have reported increased levels of miR-26a in COPD patients with chronic hypoxia [34] and in relation to the apnea process [35]. These findings highlight the importance of the hypoxemic condition in stimulating the expression of certain miRNAs. It is well known that within the mechanisms relating epigenetics and oxidative stress is the transcription factor hypoxia-inducible factor 1 α (HIF-1α) [36]. Hypoxia, or a hypoxic microenvironment, triggers the angiogenesis process, and the cellular response to hypoxia is primarily regulated by HIF-1α. Since miR-26a-5p is released from endothelial cells, its increased expression supports a relation between endothelial dysfunction and the development and progression of cancer within this group of COPD patients at increased LC risk.

The present study has some limitations. First, the present findings refer to circulating biomarkers, not to markers in lung tissue biopsies from patients, as they were not available. However, the main objective was to test the predictive value of these biological tools in non-invasive samples. Circulating miRNAs have been widely used as biomarkers due to their resistance to degradation and ubiquity. Nevertheless, further studies are needed to confirm the co-expression of the proposed miRNAs and their target mRNAs in addition to functional in vitro experiments for miRNA target regulation. Second, the number of subjects with COPD and LC included in the study may be considered small. However, this is a prospective study, where a large and very well-characterized cohort of COPD patients was included, with repeated biological samples and a long follow-up time until the development and diagnosis of LC. This fact helped obtain informative results, laying the foundations to increase the related knowledge of the potential use of miRNA-based approaches in lung cancers. Finally, the AUC values to inform the potential lung cancer risk was 0.7 for both miRNAs. This is not excessively high but could be potentiated by the addition of traditional screening tests such as chest CT or by other potentially early diagnostic markers yet to be defined. However, we do recognize that a validation of the present findings must be performed on another large prospective cohort of LC patients with an analysis of samples at several time points.

In conclusion, our study demonstrated that miR-1246 and miR-206 have potential value as non-invasive biomarkers of early lung cancer detection in COPD patients. A confirmational study on a larger prospective cohort is needed to test this signature, evaluating its use in screening and early lung cancer management.

## 4. Materials and Methods

### 4.1. Study Individuals

Ninety-nine individuals with a diagnosis of COPD recruited from the Hospital Universitario N/S de Candelaria, Tenerife, Spain, were included in this study. They are part of a cohort of 263 smokers, recruited and followed annually at this hospital as part of the BODE cohort [37], and a cohort of 3825 individuals from a lung cancer screening program (Pamplona-International early detection program, P-IELCAP) [38]. Of these patients, 13 individuals in Tenerife and 20 in the Pamplona cohort developed LC during follow-up and were enrolled in the study. Of the 33 patients with LC, 21 had two blood samples obtained: one at LC diagnosis and the other 3 years before. From the pool of COPD patients who did not develop LC over follow-up, 66 were age- and gender-matched to be used as controls. All subjects were followed annually for a mean of 72 months.

Inclusion criteria: age > 40 years, smoking history >15 pack-years, post-bronchodilator FEV1/FVC ratio < 0.70, and clinically stable for at least 6 weeks at the time of evaluation. Spirometry, lung volume, diffusion capacity for carbon monoxide, and exercise capacity were measured according to ATS-ERS guidelines [39,40]. Dyspnea was evaluated using the mMRC scale [41], the BODE index was calculated as previously described [42], and co-morbidities were quantified using the Charlson index [43]. A pulmonologist visually scored the baseline for emphysema presence using criteria established by the Fleischner Society [44]. All-cause mortality was recorded. Exclusion criteria included any other respiratory diseases and uncontrolled comorbidities such as malignancy at baseline. None of the patients had received any anti-tumor treatments at the time of sample collection.

The study was approved by the ethical committee board of Hospital Universitario N/S de Candelaria (PI 55/17, CHUNSC-2018-29). Written informed consent was obtained from all participants. The research was conducted in accordance with the Declaration of Helsinki.

### 4.2. Sample Collection

Serum samples (a total of 162) were collected from 33 COPD patients who developed LC during follow-up and 66 COPD controls without LC at the time of LC diagnosis. Of these, 21 patients had serum samples obtained 3 years before LC diagnosis that were matched with 30 controls with samples at the same timeline. The serum samples were separated from whole blood within 1 h after collection and stored at −80 °C until further use in the genetic study.

### 4.3. NGS miRNA Screening Assay

Sixteen serum samples corresponding to eight individuals (four COPD patients with LC and four COPD patients who did not develop LC during follow-up as controls) with biological samples in two timelines of analysis (at the time of LC diagnosis and 3 years before) were analyzed for the miRNA screening. RNA was isolated from 200 μL of serum using the miRNeasy Serum/Plasma Advanced Kit (Qiagen). The library preparation was carried out using the QIAseq miRNA Library Kit (QIAGEN). A total of 5µL total RNA was converted into miRNA NGS libraries. miRNAs expression screening was performed by next-generation sequencing (NGS) in an Illumina NextSeq500 platform (Exiqon A/S, Copenhagen, Denmark). The numbers of known miRNAs were calculated by counting data to relevant entries in miRBase v20 software (http://mirbase.org). The miRNA expression was expressed as Tags Per Million (TPM, the number of reads for a particular miRNA). miRNAs that were stably expressed across all samples were identified using NormFinder software [45].

### 4.4. Validation Assay: Quantitative RT-PCR for miRNA Expression

The top abundant miRNAs that resulted from the NGS screening step were validated by qRT-PCR in all study participants at two moments: at the time of LC diagnosis and 3 years before (a total of 162 serum samples). RNA was isolated from serum samples, and cDNA was synthesized by retro transcription using miRCURY LNA RT kit (Qiagen Inc., Hilden, Germany). To guarantee isolation control, the spike-ins UniSp2, UniSp4, and UniSp5 were used. In cDNA synthesis and amplification, the conjunctions of cel-miR-39-3p and UniSp6 were used as controls, as indicated by the manufacturers (miRCURY LNA RT Kit and RNA Spike-in Kit, Qiagen Inc., Hilden, Germany). The expression of the resulting significant miRNAs from the NGS screening study at the first stage was determined by an RT-PCR analysis using miRCURY LNA Sybr Green PCR Master Mix (Qiagen Inc., Hilden, Germany). A final volume of 10 μL per reaction contained 1X Sybr Green Master Mix, 200 nmol/L specific primer set (miRCURY LNA miRNA PCR assay, Qiagen Inc., Hilden, Germany), and 3 ng cDNA. All samples were performed in triplicate. The experiments were performed on a Step One Plus real-time PCR system (Applied Biosystem, Foster City, CA, USA) under the following conditions: 95 °C 15min, followed by 40 cycles of 94 °C for 15 s, 55 °C 30 s, and 72 °C 30 s. hsa-miR-486-5p and hsa-let7a-5p, which were the best candidates in the NGS screening analysis, were used as reference genes for normalization control. A non-template control was carried out in each experiment. The relative expression analysis of the target miRNAs was performed using the comparative threshold method 2−ΔΔCt [46].

### 4.5. In Silico Analysis

We integrated the miRNAs has-miR-206 and has-miR-1246 to search for potential targets in three software programs: miRBase v20 (available online: http://www.mirbase.org/), TargetScan v.7.2 (available online: http://www.targetscan.org/vert_72/), and the DIANA miRPath-v3.0 (available online: http://www.microrna.gr/miRPathv3). In the next step, the g:Profiler software (available online: https://biit.cs.ut.ee/gprofiler/gost) was used to contrast the resulting genes proposed as targets of has-miR-206 and has-miR-1246.

Functional and enrichment analyses were performed using the software’s Kyoto Encyclopedia of Genes and Genomes (KEGG) (available online: https://www.kegg.jp/) and Gene-ontology (GO) terms [47,48] through the analysis with miRTar Human software (available online: https://mirtarbase.cuhk.edu.cn/~miRTarBase/miRTarBase_2022/php/index.php).

### 4.6. Statistical Analysis

The sample was characterized using summary statistics: relative frequency of each category, 50th percentiles (5–95th) and non-scale normal, and mean ± SD, as appropriate. The comparisons between cases and controls were carried out using Pearson’s chi-square test, Mann–Whitney’s U, Wilcoxon, Fisher Exact, and Kruskal–Wallis. The correlations between variables were estimated using the Spearman or Pearson tests. miRNA expression analysis was performed at two time points: at LC diagnosis and 3 years before (median follow-up 36 months). Cases and controls were matched for age and gender.

The R statistical software package [49] and DeSeq2 software [50] were used to perform the miRNA differential expression analysis. TMM normalization of the samples was used, consisting of the trimmed mean of the M-values method based on log-fold and absolute gene-wise changes in the expression levels between samples. These fold change (FC) values were transformed to a Log scale for normalization. A miRNA was considered candidate for validation when Log2FC > 1.5. The Benjamini–Hochberg false discovery rate (FDR) algorithm was used to correct the *p*-values for multiple testing [50], where FDR is defined as the expected fraction of false rejections among those hypotheses rejected.

In the multivariate logistic regression analysis, the following covariates were included: smoking status (pack-years of smoking), BMI, and the presence of emphysema (visual score by CT scan). The sensitivity and specificity of each miRNA for the early identification of those patients who developed lung cancer were determined by receiver operator characteristic (ROC) curves. The area under the ROC curve (AUC) of each test was calculated for direct comparison.

SPSS 25.0 (IBM Co) and R software were used for all statistical analyses and two-tailed *p*-values < 0.05 were considered significant.

## Figures and Tables

**Table 1:** Main dysregulated circulating miRNAs from patients with COPD and LC vs. patients with COPD who did not develop LC during follow-up at two timelines (the time of LC diagnosis and 3 years before).

Name	Log2FC	*p*-Value	FDR
**3 years before LC diagnosis**			
hsa-miR-224-5p	2.81	**0.000003**	**0.0024**
hsa-miR-194-5p	−4.24	**0.000024**	**0.0082**
hsa-miR-206	−5.25	**0.000105**	**0.0241**
hsa-miR-26a-5p	−1.91	**0.001207**	0.2683
**At LC diagnosis**			
hsa-miR-206	−4.36	**0.000799**	**0.0553**
hsa-miR-194-5p	−2.89	**0.003618**	0.9977
hsa-miR-1246	2.25	**0.003178**	0.9977

Significant changes are defined as *p*-value < 0.001, FDR < 0.01, and Log2FC > 1.5. Abbreviations: FC, fold change; FDR, false discovery rate.

**Table 2:** Baseline demographical and clinical characteristics of COPD patients who developed lung cancer (LC) at time of diagnosis and those who did not.

Variable	COPD with LC N = 33	COPD without LC N = 66	*p*-Value
Age *	63 ± 9	63 ± 9	-
Sex (male%)	85	85	-
BMI *	28 ± 5	28 ± 5	0.719
Smoking habit (pack-year) ^‡^	64 ± 24	61 ± 27	0.631
Active smoker (%)	50	70	0.218
FEV_1_ (L) *	2.24 ± 0.74	1.67 ± 0.72	**0.001**
FEV_1_ (% pred) *	79 ± 21	62 ± 26	**0.001**
FVC (% pred) *	105 ± 20	91 ± 24	**0.005**
FEV1/FVC (% pred) *	58 ± 11	52 ± 14	**0.018**
PaO_2_ *^§^	72 ± 6	71 ± 12	0.872
K_CO_ *^§^	68 ± 25	84 ± 27	0.084
IC/TLC (%) *^§^	34 ± 8	34 ± 9	0.881
6MWD (mts) *^§^	486 ± 115	497 ± 101	0.747
Dyspnea mMRC **	0 (0–1)	1 (0–2)	0.242
BODE index **^§^	1 (0–2)	0 (0–2)	0.191
Charlson index **^§^	1 (1–1)	0 (0–1)	0.740
Emphysema (%) ^†§^	67	57	0.424
Lung cancer stage (%) I II III IV	62 9 19 10	- - - -	

* Data are presented as mean ± SD. ** Data are presented as median (25th–75thpc). ^‡^ Number of packs of cigarettes smoked per day × number of years of smoking. BMI: body mass index; FEV_1_: forced expiratory volume in one second; FVC: forced vital capacity; % pred: percent predicted; PaO_2_: partial oxygen tension; K_CO_: transfer factor coefficient of the lung for carbon monoxide, which is DL_CO_/VA; IC/TLC: inspiratory capacity to total lung capacity ratio; 6MWD: six-minute walking distance test. BODE index: body mass index, airflow obstruction, dyspnea, and exercise capacity. ^†^ Emphysema diagnosed by CT scan. ^§^ This measure was available for 11 individuals with COPD and LC. *p*-value < 0.05 (in bold).

**Table 3:** Demographical and clinical characteristics of smoker COPD patients who did and did not develop lung cancer (LC) during follow-up, three years before diagnosis.

Variable	COPD with LC N = 21	COPD without LC N = 30	*p*-Value
Age *	60 ± 9	60 ± 9	-
Sex (male%)	85	85	-
Smoking habit (pack-year) ^‡^	65 ± 20	67 ± 27	0.554
BMI	28 ± 5	28 ± 4	0.662
FEV_1_ (L) *	2.20 ± 0.78	1.51 ± 0.56	**0.004**
FEV_1_ (% pred) *	76 ± 22	54 ± 19	**0.007**
FVC (% pred) *	102 ± 21	88 ± 22	0.035
FEV1/FVC (% pred) *	58 ± 12	48 ± 13	**0.032**
PaO_2_ *^§^	74 ± 5	71 ± 10	0.457
K_CO_ *^§^	72 ± 16	84 ± 26	**0.045**
IC/TLC (%) *^§^	36 ± 8	36 ± 9	0.961
6MWD (mts) *^§^	532 ± 59	508 ± 88	0.467
Dyspnea mMRC **	0 (0–12)	1 (0–2)	0.242
BODE index **^§^	1 (0–1)	1(0–2)	0.645
Charlson index **^§^	1 (1–2)	0(0–1)	0.058

* Data are presented as mean ± SD. ** Data are presented as median (25th–75thpc). ^‡^ Number of packs of cigarettes smoked per day × number of years smoking. BMI: body mass index; FEV_1_: forced expiratory volume in one second; FVC: forced vital capacity; % pred: percent predicted; PaO_2_: partial oxygen tension; K_CO_: transfer factor coefficient of the lung for carbon monoxide, which is DL_CO_/VA; IC/TLC: inspiratory capacity to total lung capacity ratio; 6MWD: six-minute walking distance test; BODE index: body mass index, airflow obstruction, dyspnea, and exercise capacity; ^§^ 11 COPD individuals with LC were analyzed for these variables.

**Table 4:** Principal enriched KEGG pathways of predicted target genes of miR-1246 (**A**) and miR-206 (**B**).

(**A**)			
**#**	**KEGG Pathway**	**Genes**	***p*** -Value *
1	Viral carcinogenesis	PIK3CB, BAX, CDK6, TP53, KAT2b, CREB5, CCNE1	1.7095e-05
2	Oocyte meiosis	SLK, SMC1	0.0052
3	Apoptosis	CASP7, PIK3CB, BAX, BCL2, TP53, PPP3CA, CFLAR	0.0069
4	Thyroid hormone signaling pathway	MED14, PIK3CB, MED13, NOTCH2, TP53, MED1, KAT2B	0.0206
5	Central carbon metabolism in cancer	PIK2CB, TPB53, PKD1	0.0206
6	p53 signaling pathway	BAX, CDK6, TP53, TP53I3, CCNE1, CCNG2	0.0437
7	Adrenergic signaling in cardiomyocytes	PIK3CB, PPP2CA, BCL2, CALM2, ATP2B1, PPP2R1B	0.0437
8	Glioma	PIK3CB, CDK6, CALM2, TP53	0.0437
(**B**)			
#	**KEGG Pathway**	**Genes**	***p*** -Value *
1	Glycosphingolipid biosynthesis-lacto and neolacto series	FUT3, FUT9	4.395 × 10^8^
2	MicroRNAs in cancer	PCD4, MET, ROCK1, DICER1, CDC25C, CCND2, EGFR, NOTCH2, KRAS, CDK6, PRKCE, HDAC4, IKBKB, FOXP1, BCRA1, PIM1, TIMP3, NOTCH3, VMP1, MAPK1, GLS2, PDGFA	0.00101
3	Estrogen signaling pathway	ESR1, ASCY1, ATF2, CREB5, EGFR, KRAS, CALM2, SOS1, KCNJ6, IPTR3, MAPK1, PRKACB	0.0012
4	Gap junction	ADCY1, GRM5, DRD1, EGFR, KRAS, SOS1, GjA1, ITPR3, MAPK1, PRKACB, PDGFA	0.0012
5	Dorso-ventral axis formation	ETS1, CPEB1, EGFR, NOTCH2, KRAS, SOS1, NOTCH3, MAPK1	0.0043
6	Proteoglycans in cancer	ESR1, ACTB, PDC4, MET, ROCK1, CBL, FRS2, EGFR, KRAS, CBLC, TIMP3, SOS1, DDX5, IGF1, FN1, VMP1, ITPR3, MAPK1, PRKACB	0.0065
7	Glioma	CDK4, EGFR, KRAS, CDK6, CALM2, SOS1, IGF1, MAPK1, PDGFA	0.0121
8	Transcriptional misregulation in cancer	MET, PAX7, CCND2, PBX1, WT1, CDK4, H3F3A, DDX5, MAF, MAX, IGF1, PPARG, H3F3B, KMT2A, GOLPH3, MEIS1, PAX3, GRIA3, PDFGFA, COMMD3-BMI1	0.0226

Resulting pathways in relation to Tarbase v.7, TargetScan, and microT-CDS using miRPath v.3. KEGG, Kyoto Encyclopedia for Genes and Genomes. * *p* < 0.05.

## Data Availability

The datasets supporting the conclusions of this article are included within the article and its additional files.

## Supplementary Materials

The following supporting information can be downloaded at: https://www.mdpi.com/article/10.3390/ijms241512437/s1.
