# Supplementary material for: Circulating miR-206 and miR-1246 as Markers in the Early Diagnosis of Lung Cancer in Patients with Chronic Obstructive Pulmonary Disease
Source: Int J Mol Sci. 2023 Aug 4;24(15):12437. doi: 10.3390/ijms241512437 (PMC10418760; doi:10.3390/ijms241512437)
Supplement: Supplementary file 1 [file ijms-24-12437-s001.zip › ijms-2492791-supplementary.pdf]

Supplementary data:

Table S1. Baseline demographical and clinical characteristics of smokers COPD patients that develop lung cancer (LC) during follow-up, selected for miRNA expression screening by NGS.

Variable	COPD with LC N=4 3 yrs before	COPD with LC N=4 at LC diagnosis
Age*	61±7	64±7
Sex (male%)	100	100
BMI*	26±6	25±5
Smoking habit (pack-year) ‡	65±20	64±25
FEV1 (L)*	1.93±0.38	1.80±0.51
FEV1 (% pred)*	65±11	65±18
FVC (% pred)*	93±13	96±17
FEV1/FVC (% pred)*	55±7	53±9
PaO2*	75±3	72±1
KCO*	78±15	70±15
IC/TLC (%)*	32±6	31±6
6MWD (mts)*	577±53	566±59
Dyspnea mMRC**	4 (4 - 5)	4 (4 - 5)
BODE index **	1 (0 - 2)	1 (0 - 2)
Charlson index**	1 (1 - 3)	1 (1 - 1)
Emphysema (%)	75	75

*Data are presented as mean ±SD. ** Data are presented as median (25th-75thpc). ‡Number of packs of cigarettes smoked per day x number of years smoking. BMI: body mass index; FEV_1: forced expiratory volume in one second; FVC: forced vital capacity; % pred: percent predicted; PaO_2: partial oxygen tension; K_{CO}: transfer factor coefficient of the lung for carbon monoxide, which is DL_{CO}/VA; IC/TLC: the inspiratory capacity to total lung capacity ratio; 6MWD: six minutes walking distance test.

Table S2. Demographical and clinical characteristics of smokers COPD patients that did not develop lung cancer (LC) during follow-up and were selected for miRNA expression screening by NGS.

Variable	COPD without LC N=4 3 yrs before	COPD without LC N=4 At baseline
Age*	62±9	65±6
Sex (male%)	75	75
Smoking habit (pack-year) ‡	71±25	66±27
BMI	26±3	27±2
FEV1 (L)*	1.44±0.24	1.34±0.46

FEV1 (% pred)*	50±11	48±18
FVC (% pred)*	88±11	81±22
FEV1/FVC (% pred)*	44±8	45±12
PaO2*	72±8	75±2
KCO*	83±20	77±19
IC/TLC (%)*	32±5	30±14
6MWD (mts)*	552±41	511±94
Dyspnea mMRC**	3 (2-3)	3 (3-4)
BODE index **	1 (1-3)	1 (0-5)
Charlson index**	1 (0-2)	1 (0-2)

*Data are presented as mean ±SD. ** Data are presented as median (25th-75thpc). ‡Number of packs of cigarettes smoked per day x number of years smoking. BMI: body mass index; FEV_1: forced expiratory volume in one second; FVC: forced vital capacity; % pred: percent predicted; PaO_2: partial oxygen tension; K_{CO}: transfer factor coefficient of the lung for carbon monoxide, which is DL_{CO}/VA; IC/TLC: the inspiratory capacity to total lung capacity ratio; 6MWD: six minutes walking distance test.

Table S3. Targeted enriched Gene Ontology (GO) biological process shared by miR-1246 and miR-206.

GO Category (Biological process)	p-value*
1. Cellular nitrogen compound metabolic process	1.59195466316e-35
2. Biosynthetic process	2.26848315643e-21
3. Cellular protein modification process	1.03264028534e-16
4. Gene expression	4.37638703546e-12
5. Mitotic cell cycle	1.23223160695e-11
6. Symbiosis, encompassing mutualism through parasitism	1.23223160695e-11
7. Viral process	1.17714015187e-10
8. Blood coagulation	1.59118929907e-09
9. Cellular component assembly	3.46220305987e-09
10. Macromolecular complex assembly	2.6511295871e-08
11. Protein complex assembly	1.80982122582e-07
12. Fc-epsilon receptor signaling pathway Inositol phosphate metabolic process	2.74256667512e-07
13. Membrane organization	7.72426792997e-07
14. Neutrophin TRK receptor signaling pathway	1.60507581507e-06
15. Cell death	3.36236599369e-06

*p-value<0.001

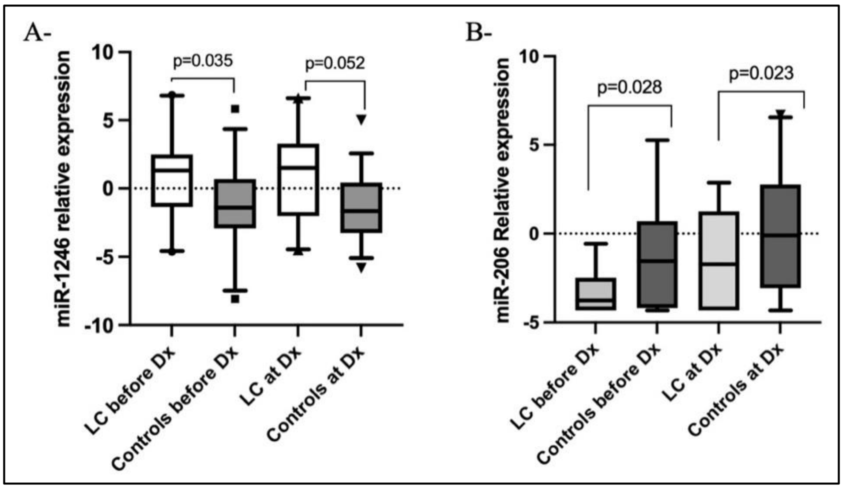

Figure S1. miR-1246 and miR-206 relative expression in COPD patients with LC in contrast to COPD controls at the time of LC diagnosis and three years before.

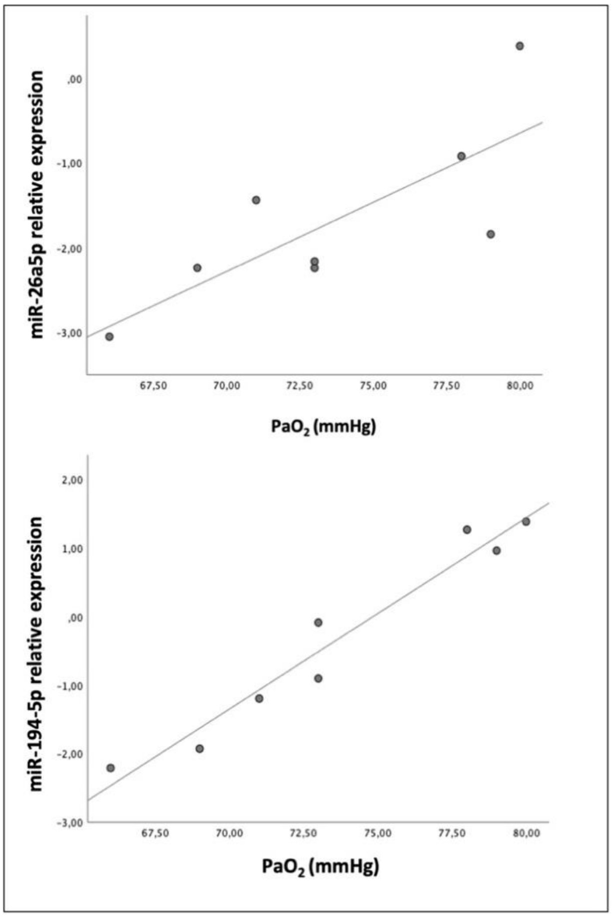

Figure S2. Correlation between miR-26a-5p and miR-194-5p and the levels of PaO2 in patients with COPD before LC diagnosis.
